# Pharmacomechanical Thrombolysis in the Management of Paget-Schroetter Syndrome

**DOI:** 10.1155/2013/214804

**Published:** 2013-02-11

**Authors:** Elli Papantoniou, Luke Morgan-Rowe, Edward Johnston, Duncan Brennand, Jowad Raja, Julian Hague

**Affiliations:** Multidisciplinary Endovascular Team, University College London Hospitals, 235 Euston Road, London NW1 2BU, UK

## Abstract

Paget-Schroetter syndrome (PSS) is a rare form of thoracic outlet syndrome caused by axillosubclavian vein thrombosis which typically presents in healthy young adults. Prompt therapy, traditionally by means of catheter-directed thrombolysis (CDT) prior to definitive surgery, can prevent the subsequent onset of postthrombotic syndrome (PTS) and considerable disability. As CDT is associated with major haemorrhage and high overall treatment cost, pharmacomechanical thrombectomy (PMT) seems to be an attractive alternative which combines pharmacological thrombolysis with mechanical clot disruption. The Trellis-8 peripheral infusion catheter is an example of such a treatment which provides topical thrombolysis in an isolated zone. We describe the use of the Trellis-8 PMT system in the successful management of three patients with PSS.

## 1. Introduction

Paget-Schroetter syndrome (PSS) is the consequence of axillosubclavian vein thrombosis arising from thoracic outlet syndrome. Typically occurring in active young adults, it is the consequence of repetitive micro trauma to an abnormally stenotic subclavian vein and results in acute or acute-on-chronic thrombosis [1].

Serious sequelae can result from untreated PSS, including pulmonary embolism (PE) [2] and postthrombotic syndrome (PTS) [3]. Prompt intervention is therefore necessary and must combine the removal of thrombus with correction of venous stenosis: usually by means of thrombolysis, percutaneous venoplasty, and surgical decompression of the thoracic outlet [1].

Catheter-directed thrombolysis (CDT) has become the standard of thrombolytic care in many institutions [1, 4, 5] but is associated with several disadvantages including major systemic haemorrhage [4, 6] and high overall treatment cost [7]. Pharmacomechanical thrombectomy (PMT) addresses these disadvantages by combining mechanical clot disruption and pharmacological thrombolysis within an isolated zone. Although PMT has been investigated in lower-limb DVT [5], only a few reports are dedicated to use in upper-limb DVT [8–10].

PMT devices include the AngioJet system and the Trellis-8 device (Covidien, Santa Clara, CA) [5, 10, 11]. Whilst the AngioJet system employs a power-pulse spray technique, the Trellis-8 is a peripheral infusion catheter designed to isolate the thrombolytic zone with proximal and distal compliant balloons, confine the mechanical disruptive process, and prevent spread of the thrombolytic agent. Following physical clot disruption using a wire oscillating at high frequency within the vein, the resultant product (including the thrombolytic) is aspirated into a syringe prior to balloon deflation [5].

We describe three cases of PSS managed in our institution with PMT via the Trellis-8 device prior to first rib resection.

## 2. Case Report

### 2.1. Case 1

An 18-year-old male presented to the emergency department with a three-day history of pain, swelling, and discolouration in the left arm and clinical diagnosis of upper limb deep vein thrombosis was made.

Catheter venography demonstrated occlusion of the left subclavian vein around the first rib with extensive thrombus and collateralised flow, typical of PSS. The brachiocephalic vein and superior vena cava were patent. 

**Figure 1:**
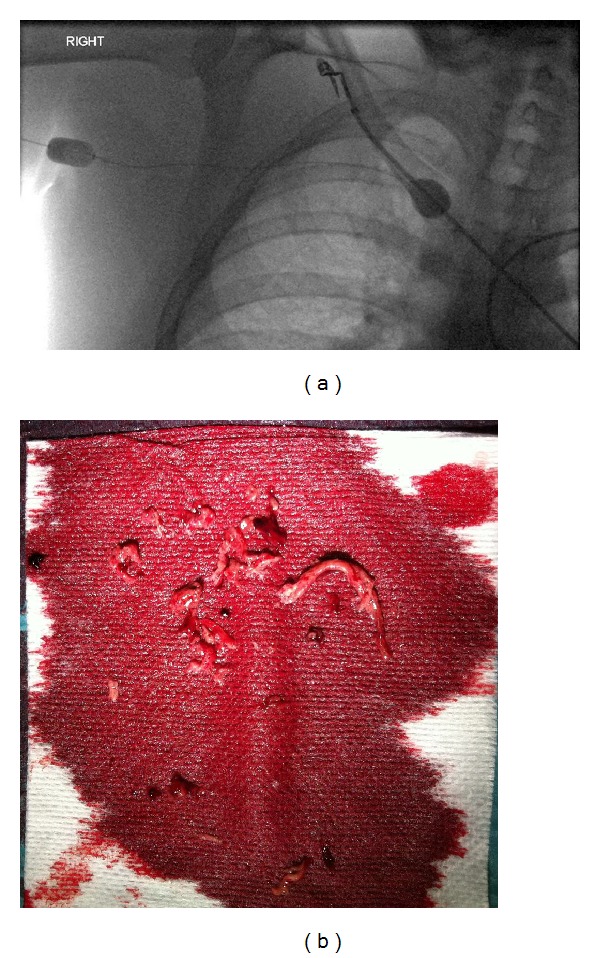
(a) Fluoroscopic image showing the distal compliant balloon of the Trellis-8 device inflated in the brachiocephalic vein and the proximal balloon inflated in the subclavian vein. (b) Aspirated thrombus following PMT. There is a mix of fresh and chronic thrombi.

Under local anaesthesia, the brachial vein was punctured using ultrasound guidance and a 5 French micropuncture kit (Cook, Bloomington, IN). An 8 French, 9 cm Brite-tip sheath (Cordis, Miami, FL) was placed in the brachial vein distal to the thrombus, and the occlusion was crossed with a hydrophilic 0.035^*″*^ wire (Terumo Corp, Japan). 5000 units of unfractionated heparin were given via the sheath. An 8  French, 15 cm Trellis-8 device (Covidien, Mansfield, MA) was placed across the lesion and the balloons inflated. The distal balloon was positioned in the brachiocephalic vein and the proximal balloon in the axillary vein (Figure 1(a)), isolating the thrombus from the systemic circulation. The Trellis procedure was performed for 10 minutes with concomitant infusion of 10 mg of recombinant tissue plasminogen activator (rT-PA). The product was aspirated through the device (Figure 1(b)) and into a 6  French, 55 cm Flexor sheath (Cook, Bloomington, IN).

Venoplasty was then performed on the tight, irregular stenosis of the subclavian vein as it crossed the first rib using an 8 × 40 mm fox plus balloon catheter (Abbott, IL). An intravenous unfractionated heparin infusion was commenced at 1000 u per hour, and the patient was listed for emergency first rib resection (within 2 hours). Our procedure was well tolerated by the patient with no requirements for sedoanalgesia.

Repeating venography the following day showed a residual stenosis which responded to 10 mm balloon venoplasty. Subsequent duplex studies at 4 days and 6 weeks showed a patent vein with no residual stenosis. 

At discharge, anticoagulation using warfarin was commenced to achieve a target international normalised ratio (INR) of between 2.5 and 3.5. By six months, the patient was well with minimal swelling in the arm, anticoagulation was discontinued, and he has resumed his normal activities.

### 2.2. Case 2

A 26-year-old female presented to the emergency department with a history of sudden-onset left arm pain and swelling. Past medical history included recent commencement of the oral contraceptive pill and a first-degree relative with DVT.

**Figure 2:**
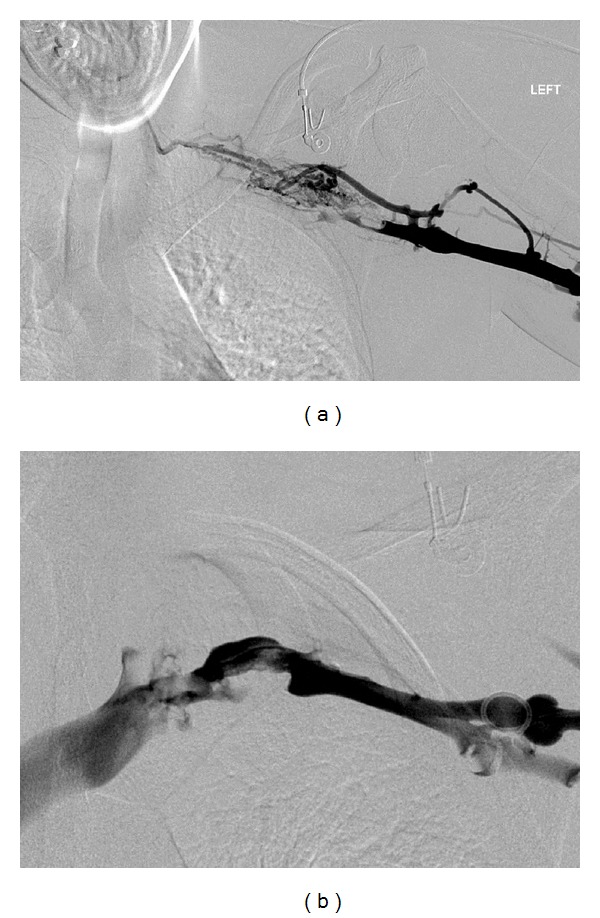
(a) Digital subtraction venogram showing occlusive thrombus in the axillary and subclavian veins with extensive collateralisation. (b) Digital subtraction venogram following PMT. Flow through the axillary and subclavian veins has been restored with minimal residual thrombus. A stenosis is seen in the first rib.

Venography revealed occlusive thrombus in the axillary and subclavian vein with extensive collateralisation (Figure 2(a)). PMT was performed using the Trellis-8 device (method as described previously) and 10 mg of rT-PA. A venous stenosis, typical of PSS, was demonstrated at the level of the first rib (Figure 2(b)), and the patient was transferred to the operating theatre for first rib resection.

The arm swelling rapidly reduced, and the patient was discharged on full anticoagulation. She has been lost to followup.

### 2.3. Case 3

**Figure 3:**
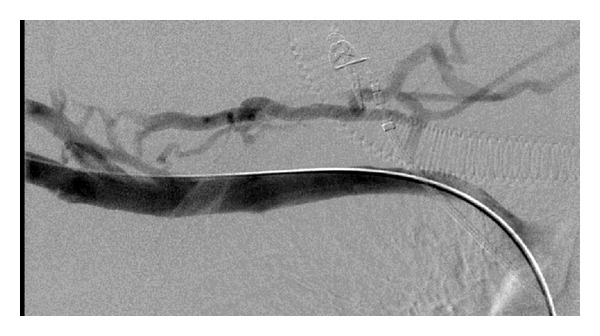
Digitial subtraction venogram following PMT, showing complete recanalisation of the occluded subclavian vein.

A 57-year-old male with positive antiphospholipid antibodies developed acute right arm swelling following strenuous exercise. Venous Doppler ultrasound demonstrated occlusive thrombus in the right subclavian and brachial veins. He underwent PMT with the Trellis-8 device using 10 mg rT-PA. Angiographic results were excellent (Figure 3) and the patient was commenced on lifelong warfarin for the underlying thrombophilia under haematological advice.

The patient was discharged following resolution of his symptoms and remain well at 6-month followup. 

## 3. Discussion

Paget-Schroetter syndrome is a consequence of an anatomically deranged thoracic outlet, most commonly caused by congenital bands, cervical ribs, scalenus tendon hypertrophy, or variant insertion of the costoclavicular ligament. Consequently, normal upper-limb movements provide insult to the subclavian vein which responds with venous intimal hypertrophy, chronic inflammation, peri-venular fibrosis and acute or acute-on-chronic thrombosis.

Presenting features include upper-limb pain, heaviness, swelling and cyanosis and are often preceded by a history of strenuous exercise or trauma. The influence of inherited thrombophilic disorders on this condition is unresolved [1].

Postthrombotic syndrome (PTS) is a complication of venous thrombosis and has recently been recognised in upper-limb DVT. With an overall incidence of 7%–46% [3], it comprises a constellation of chronic pain, paraesthesia, heaviness, and functional limitation which can significantly impact patients' quality of life.

Although jugulosubclavian vein bypass and patch venoplasty have been described in the management of PTS [12], supportive measures are mainstay. As such, the aggressive and early treatment of PSS is paramount. Management of PSS usually incorporates thrombolytic therapy, percutaneous venoplasty, and surgical decompression of the thoracic outlet [1, 8, 9]. The need for and timing of surgery is debated, with some investigators reserving surgery for those with persistent or recurrent symptoms following thrombolysis, and others preferring early decompression for all patients [1].

Catheter-directed thrombolysis has become standard practice in many institutions prior to surgical management. However, this often requires Intensive Care Unit (ICU) admission, regular blood sampling and repeated angiography to assess treatment response. Median time to complete resolution in the upper-limb DVT averages 24–48 hours [8, 10], and CDT alone may not provide complete treatment [7] with major haemorrhage as a well-recognized complication [4, 6]. To this end, costs of CDT can be considerable. 

Pharmacomechanical thrombectomy obviates the need for ICU admission and eliminates the systemic effects of thrombolytic agents. In particular, the Trellis-8 system was shown to have a significantly greater rate of clot lysis (93%) in comparison with CDT (79%) in a recent meta-analysis of published trials [7]. Furthermore, the time to lysis was substantially shorter with Trellis-8 (2 minutes versus 24 hours).

As Trellis-8 requires lower doses of a thrombolytic agent which is confined to the treatment zone, it is associated with lower rate of haemorrhagic complications, a shorter time in the angiography suite, and minimal (if any) time in the ICU. Consequently, Trellis-8 has been shown to confer an economic advantage to CDT [7, unpublished data courtesy of Dr. G. O'Sullivan, Galway, Ireland].

The Trellis-8 catheter has several advantages over other PMT devices. Firstly, there is no significant systemic thrombolysis evidenced by laboratory markers (e.g., fibrinogen) [11] which decreases the risk of further haemorrhage and permits other interventions which may be required all around thrombolysis. This is particularly relevant to patients with PSS where immediate surgery is often required. Secondly, by isolating and extracting thrombus, there is probably a reduced risk of the pulmonary or distal embolic complications described with the use of other PMT devices [11, 13].

However, certain issues require careful consideration when using the Trellis-8 infusion catheter. Firstly, although thrombolytic doses and the risk of haemorrhage are reduced, the overall safety of Trellis-8 in upper-limb PMT has not been formally evaluated in clinical trials. Additionally, concerns have been raised regarding the systemic effects of PMT, for example, the induction of bradyarrhythmias [14]. Furthermore, the cost advantages of Trellis-8 have only been proven in lower-limb DVT. Economic advantages of its use in upper-limb DVT where the extent and burden of thrombosis may be less are yet to be evaluated. Finally, although the introduction of large calibre sheaths into brachial veins may raise concerns of technical feasibility and complication rate, this has not been an issue in our experience.

## 4. Conclusion

PSS is a condition of young, healthy, and active patients with potentially serious long-term sequelae if untreated or undertreated. We have described three cases of successful treatment of PSS incorporating the Trellis-8 peripheral infusion catheter. PMT has several advantages over CDT in the management of PSS as a precursor to surgery, and the Trellis-8, which specifically provides isolated PMT, seems to be safe, effective and well tolerated in this patient population. This paper seeks to assist in raising the awareness of the clinical and venographic features of PSS and provide additional management options for the endovascular specialist.
